# Supplementary material for: The Relationship and Influencing Factors between Endangered Plant Tetraena mongolica and Soil Microorganisms in West Ordos Desert Ecosystem, Northern China
Source: Plants (Basel). 2023 Feb 25;12(5):1048. doi: 10.3390/plants12051048 (PMC10005437; doi:10.3390/plants12051048)
Supplement: Supplementary file 1 [file plants-12-01048-s001.zip › plants-2116297-supplementary.pdf]

Supporting material

Table S1 The composition of functional groups of fungi in Tetraena mongolica community.

Functional Group types	Proportion
Saprotroph	0.38
Pathogen	0.28
Ectomycorrhizal	0.09
Endophyte	0.07
Parasite	0.06
Endomycorrhizal	0.05
Arbuscular Mycorrhizal	0.05
Epiphyte	0.01

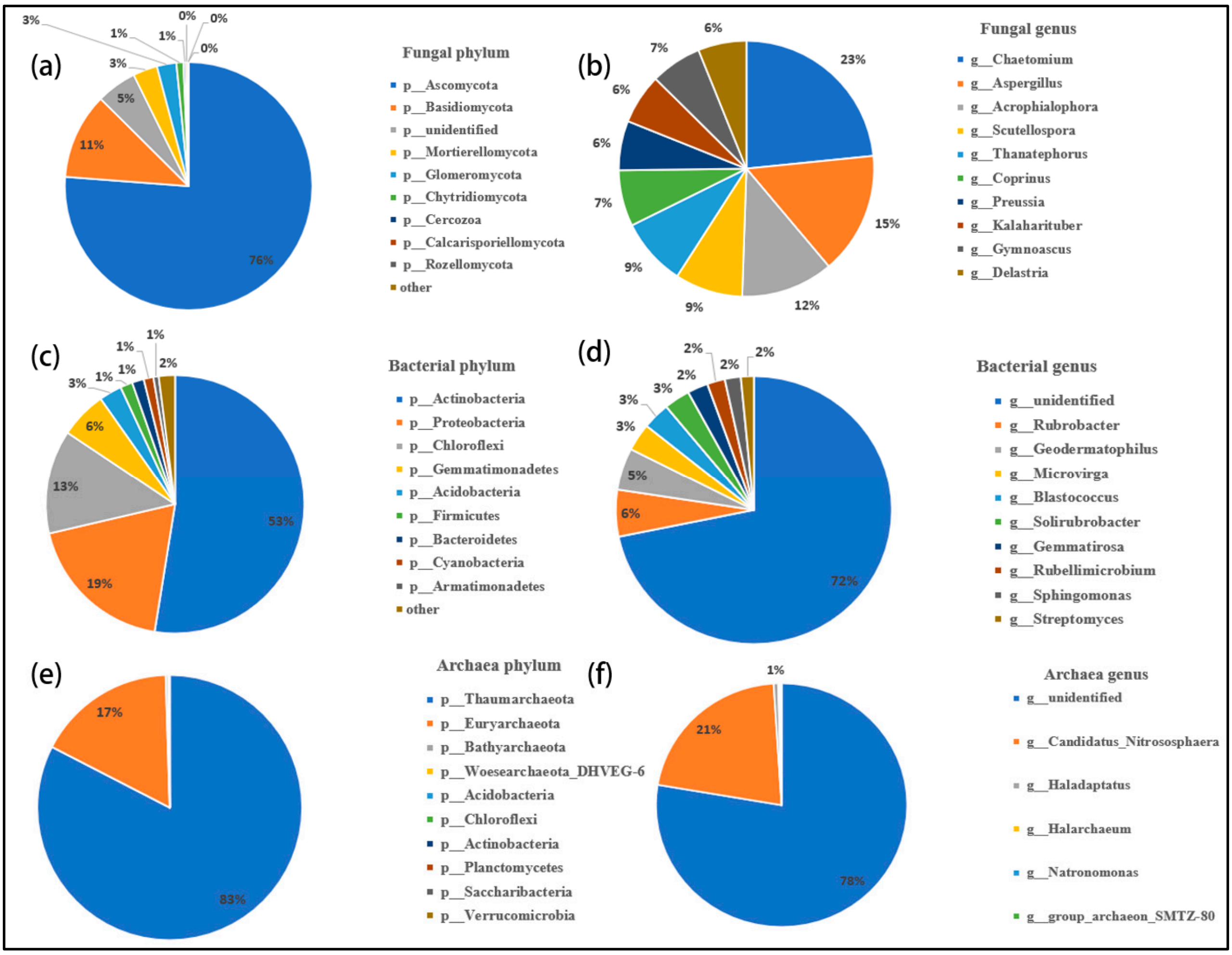

Figure S1 The main phylum and genus composition of fungi, bacteria and archaea in the *Tetraena mongolica* community. Figure a–b shows the composition of phyla and genus of fungi, figure c–d shows the composition of phyla and genus of bacteria, and figure e–f shows the composition of phyla and genus of archaea.
